# Impact of *Crocus sativus* L. on Metabolic Profile in Patients with Diabetes Mellitus or Metabolic Syndrome: A Systematic Review

**DOI:** 10.3390/nu12051424

**Published:** 2020-05-14

**Authors:** Parthena Giannoulaki, Evangelia Kotzakioulafi, Michail Chourdakis, Apostolos Hatzitolios, Triantafyllos Didangelos

**Affiliations:** 1 Department of Nutrition and Dietetics, University General Hospital of Thessaloniki AHEPA, 54621 Thessaloniki, Greece; nenagian@yahoo.com; 2 Diabetes Center, 1st Propeudetic Department of Internal Medicine, School of Health Sciences, Medical School, Aristotle University of Thessaloniki, 54621 Thessaloniki, Greece; evelinakotzak@hotmail.com (E.K.); chatzito@auth.gr (A.H.); 3 Laboratory of Hygiene, Social & Preventive Medicine and Medical Statistics, Department of Medicine, School of Health Sciences, Aristotle University of Thessaloniki, 54124 Thessaloniki, Greece; mhourd@gapps.auth.gr

**Keywords:** diabetes mellitus, *Crocus sativus* L., saffron, crocin, picrocrocin, safranal, dyslipidemia, hyperglycemia

## Abstract

Background: Experimental studies demonstrated a positive effect of administration of *Crocus sativus* L. (saffron) and its bioactive ingredients on metabolic profile through their antioxidant capacity. Purpose: To determine if the use of saffron in humans is beneficial to patients with diabetes mellitus (DM) or metabolic syndrome (MS). Methods: This systematic review includes 14 randomized control trials that investigated the impact of saffron administration and its bioactive ingredient crocin on the metabolic profile of patients with DM, MS, prediabetes, and coronary artery disease. We documented the following clinical outcomes: fasting blood glucose (FBG), glycated haemoglobin (HbA1c), total cholesterol, low-density lipoprotein (LDL) cholesterol, high-density lipoprotein (HDL) cholesterol, triglycerides, systolic, and diastolic blood pressure. Results: Eight studies examined the efficacy of saffron in patients with DM, four with the metabolic syndrome, one with prediabetes and one with coronary artery disease. A favorable effect on FBG was observed. The results regarding blood lipids and blood pressure were inconclusive in the current review. Conclusions: According to the available limited evidence, saffron may have a favorable effect on FBG. Many of the studies in the reviewed literature are of poor quality, and more research is needed in this direction to confirm and establish the above findings.

## 1. Introduction

Diabetes mellitus (DM) is a disorder of the metabolism of carbohydrates, proteins, and lipids, and its principal characteristic is hyperglycemia due to lower secretion or lack of insulin. Prolonged uncontrolled DM leads to certain microvascular complications such as nephropathy, neuropathy, retinopathy, and macrovascular complications, such as cardiovascular disease and stroke. Poor glycemic control is the main cause worldwide of end-stage chronic kidney disease, amputations, and blindness [1]. Due to DM’s increasing prevalence globally, it has already become a major cause of cardiovascular morbidity and mortality. Moreover, costs for DM treatment and its complications are a substantial economic burden for many countries. Furthermore, patients with DM complications have a decreased quality of life and life expectancy [2,3].However, optimal control of plasma glucose and lipid concentrations can reduce the incidence of DM-related complications [4], but optimal metabolic control is difficult to achieve and maintain over time, especially in type 1 DM patients. 

Medical nutrition therapyis the cornerstone of the prevention and management of DM. Appropriate healthy eating habits, including low-carbohydrate, low glycemic load, and high-fiber diets with regular physical activity, and adequate sleep duration are associated with optimal glycemic control and achieving ideal body weight. Moreover, the favorable effects of the most popular glucose-lowering agents, dipeptidyl peptidase-4 (DPP-4) inhibitors, and sodium-glucose cotransporter 2 (SGLT-2) inhibitors are significantly affected by body mass index (BMI) and dietary patterns [5].

Although there are many treatment options for DM, the high cost of DM medication and its side effects have led researchers to investigate alternative treatment options. Among those is the use of saffron due to its high antioxidant capacity, which in many studies has been shown to exert a protective action against cell and tissue damage.

The plant *Crocus sativus* L. is a bulbous and perennial plant with red stigmas. Its red stigmas in dried form is the spice commonly known as saffron or crocus [6]. Saffron is produced mainly in Greece, Iran, and India. The plant extract contains potential pharmacological active ingredients, such: crocins (mono- and diglycosylic esters of dicarboxylic acids, crocetin), picrocrocin and safranal. The primary active ingredients are crocins (approximately 10% of the total content) [7]. High-quality saffron consists of approximately 30% crocins, 5–15% picrocrocin, and often 2.5% volatile compounds, one of which is safranal. Greek saffron, known as Greek red saffron, has the highest concentration of the above ingredients [8].

Experimental studies in animals have shown that saffron demonstrates antidiabetic and antioxidant properties. These studies showed that saffron and its bioactive components have a positive impact on hyperglycemia due to the improvement of fasting blood glucose (FBG) on serum insulin and HbA1c levels, advanced glycation end products (AGEs) production [9,10,11,12,13,14] and insulin sensitivity [15,16,17].

Moreover, in vitro and in vivo studies demonstrated the potential of saffron and its constituents in reducing the level of total serum cholesterol(t-chol), low-density lipoprotein cholesterol (LDL-c) and triglycerides (TG) and improving the levels of high-density cholesterol (HDL-c) and the ratio of LDL-c/HDL-c in healthy, diabetic and dyslipidemic animals [9,18,19,20,21,22,23]. 

In addition, it has been reported that the aqueous extract of saffron and its two compounds, crocin and safranal, can reduce mean arterial blood pressure in animals in a dose-dependent manner [24,25,26,27].

Research indicates that saffron and its constituents have a significant role in the inhibition and regression of atherosclerosis by preventing apoptosis in animal models [28,29,30,31,32,33,34,35] and improve adverse results from myocardial injury by significantly reducing the levels of lactate dehydrogenase (LDH), creatine kinase (CK), malondialdehyde(MDA)and increasing the level of superoxide dismutase(SOD) in rat myocardial ischemia model [36,37,38,39,40,41,42].

The balance between the removal and the production of cellular reactive oxygen species (ROS) is defined as the “redox state”. There is no doubt that ROS are increased in the setting of DM. Many studies have tried to eliminate increased production with various agents (vitamins, antioxidants, etc.), but their results were inconclusive, possibly because we do not know the exact level of the removal of ROS. Some levels of ROS are needed for cell functionality. On the other hand, a dietary pattern rich in antioxidants, as is the Mediterranean dietary pattern, could provide a considerable reduction in cardiovascular risk and may be of particular benefit to subjects with diabetes mellitus [43]. 

Overall, a growing body of evidence has focused on the medicinal properties of saffron as an antidiabetic, hypolipidemic, anti-hypertensive, and cardioprotective agent in animals. However, previous work that examines the above properties in patients with DM and metabolic syndrome (MS) is limited. This review aimed to present and assess the results of relevant studies, regarding the impact of saffron and its bioactive components on the metabolic profile of patients with DM and the MS. Also, existing gaps in the available literature are discussed, and potential areas of future research are proposed.

## 2. Materials and Methods

**Figure 1:**
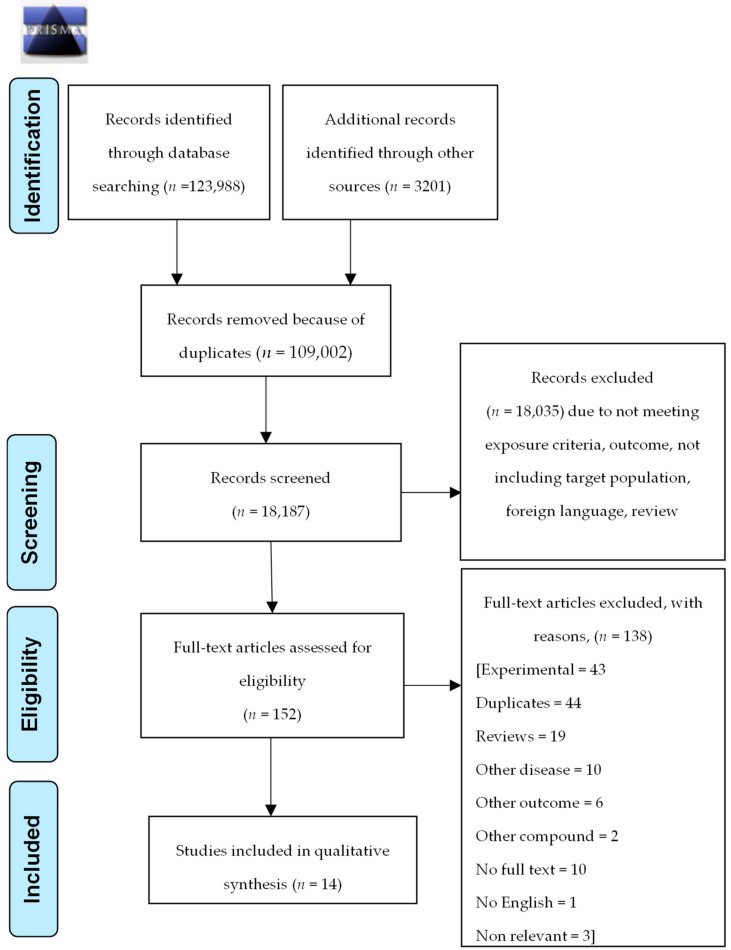
PRISMA flow diagram.

This systematic review was conducted following the guidelines of Preferred Reporting Items for Systematic Reviews and Meta-Analyses (PRISMA) (checklist is included as Supplementary Materials) and PRISMA flow diagram is presented in Figure 1.

### 2.1. Literature Search Strategy

Two reviewers (PG and EK) independently performed an extensive literature search from July 2019 until September 2019 from the following databases: MEDLINE (via PubMed), Scopus (Science Direct), Cochrane Library Database of Systematic Reviews, Google Scholar and Clinicaltrials.gov. A final search was performed in January 2020 to identify any new publications. No filter was used during the search process. A combination of keywords and Boolean logic was used to search in all databases including “diabetes mellitus” or “antidiabetic” or “hyperglycemia” or “hypertension” or “metabolic syndrome” or “dyslipidemia” or “hyperlipidemia” or “hypoglycemia” or “atherosclerosis” or “macrovascular diabetic complications” or “microvascular diabetic complications” or “cardiovascular disease” or “myocardial injury” or “insulin sensitivity” or “insulin resistance” and “crocus sativus” or “crocin” or “picrocrocin” or “saffron” or “safranal”. Also, an additional search was performed in PubMed using Medical Subject Headings (Mesh): “Diabetes Mellitus”[Mesh] AND ((((“Crocus”[Mesh]) OR “crocin” [Supplementary Concept]) OR “picrocrocin” [Supplementary Concept]) OR “safranal” [Supplementary Concept].

### 2.2. Types of Studies and Eligibility Criteria

Reviews, meta-analyses, experimental studies in vitro, and in vivo and clinical trials that were ongoing or had not published results yet were excluded from the search process. Additionally, any study for which full text was not retrieved or was not available in English language was also excluded. Furthermore, studies that examined different outcomes other than the metabolic profile or studied other diseases or other herbal compounds were excluded too. Only randomized controlled trials (RCTs) in human subjects with DM and MS were included. Studies included had biochemical metabolic markers, such as t-chol, HDL-c, LDL-c, TGlevels, FBG, HbA1c, waist circumference (WC), systolic and diastolic blood pressure (SBP and DBP) as an outcome.

### 2.3. Data Collection and Extraction

Abstracts of full texts were independently read by two reviewers (PG and EK) to assess their eligibility for this review and evaluated according to the eligibility criteria. Information and results that were of interest in each study were reported in a standardized manner. The extracted data from each study included the following characteristics: (I) citation, author, publication year and purpose; (II) inclusion and exclusion criteria; (III) type of intervention and type and amount of substance used; (IV) sample size; (V) baseline and after intervention metabolic variables; (VI) if dietary and physical activity assessments were used; (VII) reported conclusions; (VIII) funding sources and (IX) conflict of interest statement. 

### 2.4. Study Quality and Risk of Bias Assessment in Included Studies

The quality of each study was independently assessed by two reviewers (PG and EK) using Cochrane Collaboration’s tool for assessing the risk of bias in RCTs. Assessment of each study was entered into the software Review Manager 5.3. Critical assessment of several domains was performed, including selection bias, performance bias, detection bias, attrition bias, reporting bias, and other biases. Each assessment was characterized as either low or high or unclear risk. Two figures were generated by Review Manager to demonstrate all assessments of the included studies regarding risk of bias. There was no blinding of reviewers regarding study authors and journal. Consensus was reached for all studies included. 

### 2.5. Data Synthesis and Analysis

Narrative synthesis and analysis of the data of each study was made. No meta-analysis was performed due to the high heterogeneity regarding study design and reported outcomes between included studies. Wherever there were two intervention groups in a study, the statistics of these two groups were combined in one, using the handbook Cochrane formula for combining two groups. 

## 3. Results

### 3.1. Characteristics of the Studies Included in the Review

Fourteen studies met the eligibility criteria for this systematic review. All studies were randomized placebo-controlled trials, eight in patients with DM [44,45,46,47,48,49,50,51], four with MS [52,53,54,55], one with prediabetes [56], and one with coronary artery disease [57]. All of them had at least one intervention arm with oral administration of saffron extract or crocin and a placebo arm. Reported outcomes were changes in metabolic variables such as FBG, HbA1c, t-chol, HDL-c, LDL-c, TG, WC, SBP, and DBP (Table 1).

### 3.2. Risk of Bias Assessment

**Figure 2:**
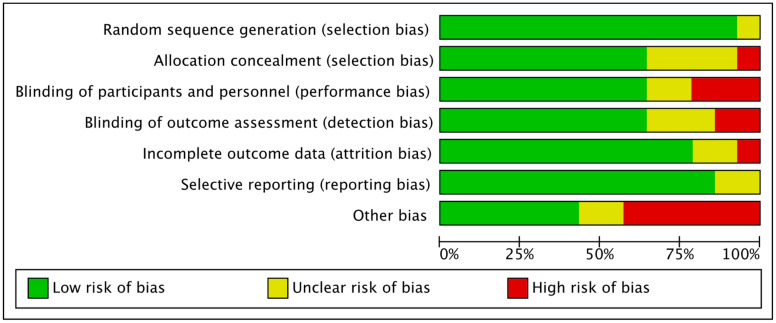
Risk of Bias assessment summary.

**Figure 3:**
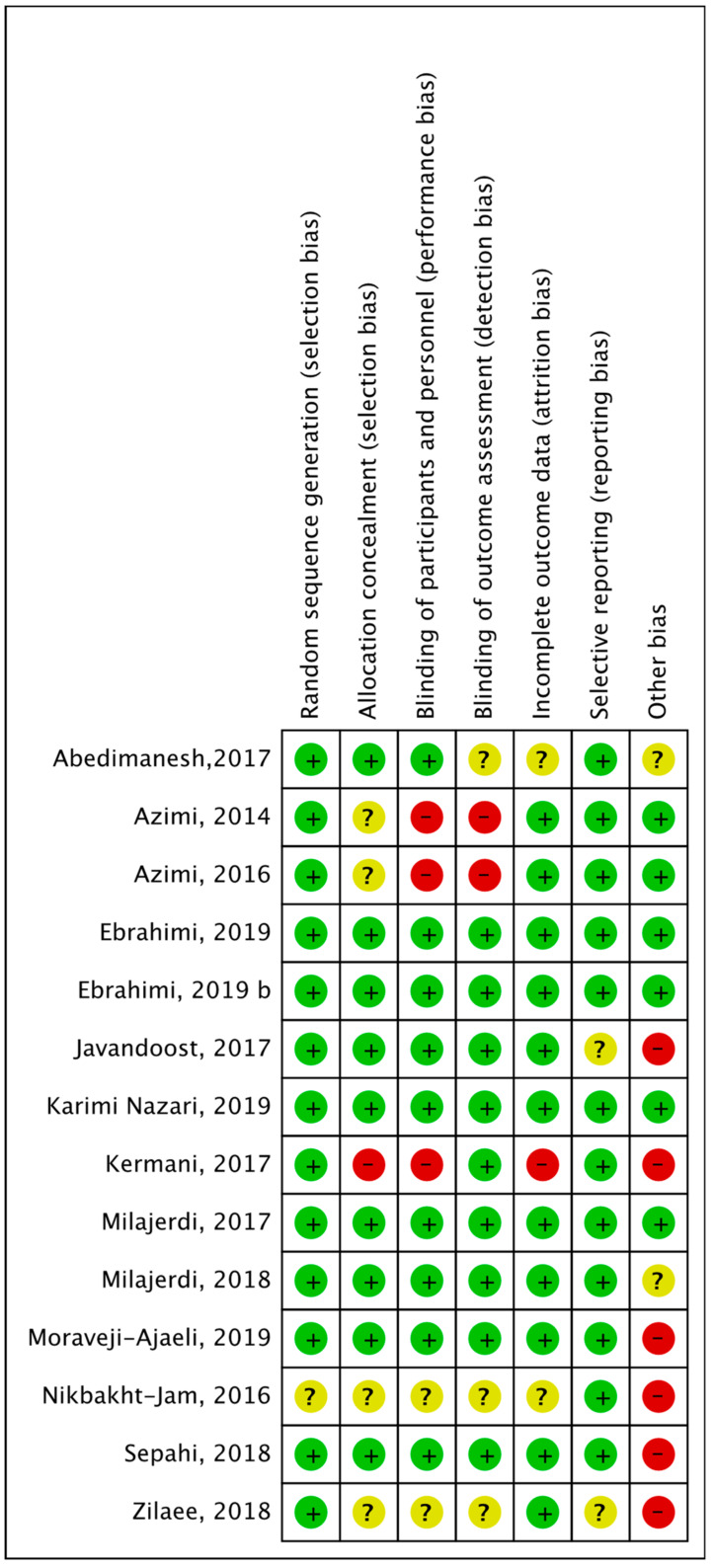
Risk of bias assessment summary for each included study.

All studies reported the randomization technique that was followed. One study [53] reported allocation methods that imply blinding of personnel could have been broken, so this was considered as of high risk of bias in allocation concealment. Studies that provided insufficient information about allocation methods were considered asexhibiting unclear risk of bias [44,45,54,55]. Two single- blinded studies [44,45] were reported as being of high risk, and other studies [53,54,55] were characterized as demonstrating unclear risk as their blinding method was not mentioned, although reported as double-blinded. In relation to blinding of outcome assessment, five studies [53,54,55,57] were alsocharacterized as having an unclear risk because they were mentioned as double-blind but did not provide sufficient details on the method and two [44,45] were judged as exhibiting a high risk because they were single-blinded. All others were assessed as low-risk studies providing adequate evidence. Two studies [54,57] were considered of unclear risk because they did not address what missing data they had, and one [53] was of high risk due to high percentage of dropout (20%) that may have influenced their results. Except for one [55], all studies prespecified their primary and secondary endpoints with trial registration, so their judgement for selective reporting was considered low risk. Other biases were considered, such as not reporting assessment of diet and physical activity, if the company that produced the product was funding the trial or if there were any methodological issues that were not included in the mentioned bias. Figure 2 shows the risk of bias assessment across all included studies and Figure 3 shows the risk of bias summary, including assessment of each risk item of all included studies.

### 3.3. Glycemic Control

Only three studies [49,50,56] out of 10that examined FBG as a primary outcome showed a significant reduction in FBG (*p* < 0.001; *p* = 0.013; *p* = 0.005 respectively) after oral administration of saffron or crocin versus placebo (Table 2). Out of those three studies, only Karimi et al. demonstrated also a significant reduction (*p* < 0.005) in HbA1c. Moreover, Sepahi et al. showed a significant reduction (*p* = 0.024) of HbA1c only in one of the two intervention arms (15 mg crocin) compared to the placebo arm (Table 3). 

### 3.4. Lipid Profile

Total cholesterol concentration was significantly reduced after the intervention in only three studies [44,50,53] out of 11. It is notworthy, that Kermani et al. [53] found a significant reduction in t-cholconcentration both in the intervention ( *p* < 0.001) and the placebo groups(*p* = 0.01) at the end of the study, but there was not a significant difference in the magnitude of reduction between the two groups (Table 4). On the other hand, Azimi et al. [44] and Moravej-Aleali et al. [50] found significant differences in the reduction of t-cholconcentration (*p* = 0.004; *p* = 0.014 respectively) and LDL-concentration (*p* = 0.01; *p* = 0.014 respectively) between their two groups. Zilaee et al. [55] showed a significant reduction in LDL-concentration after the intervention (*p* = 0.03)between the two groups (saffron group vs. placebo group), whilst Nikbakth [54] showed a reduction only between baseline and post intervention in the crocin arm (*p* = 0.02),whereas the magnitude of reduction was not significant between the two arms (Table 5). Azimi et al. [44], found a significant difference in HDL-c (*p* = 0.001) as well. In a study by Javandoost et al. [52], HDL-c increased significantly after the intervention both in the crocin and the placebo arm (*p* = 0.004; *p* < 0.001 respectively) (Table 6). A significant post-intervention reduction (*p* < 0.003) was seen in TG only byKermani et al. [52], and only in the crocin group (Table 7).

### 3.5. Other Components of Metabolic Syndrome (MS)

Ebrahimi et al., [46] found a significant difference in the reduction (*p* = 0.005) of SBP between the two groups (Table 8) but not of DBP (Table 9).Furthermore, a significant reduction in WC was observed only in the study of Ebrahimi et al. [47] ( *p* < 0.001) in the saffron group versus placebo (Table 10). 

## 4. Discussion

This systematic review includes 14RCTs that investigated the impact of saffron administration and its bioactive ingredient crocin on the metabolic profile in patients with DM and MS. In particular, the following clinical outcomes: FBG, HbA1c, t-chol, LDL-c, HDL-c, TG, WC, SBP, and DBP were documented.

Some of the studies we examined provided significant differences in the lipid profile either between the intervention and placebo group or within the groups [44,53,54,55]. These results were very inconclusive and the studies they came from had a non-favorable risk of bias, so they were excluded from our conclusions.

According to the risk of bias assessment, eight studies [46,47,48,49,50,51,52,56] were not of low quality (i.e., based on Cochrane criteria for considering a study of low quality: it should have a judgement on high risk of bias ≥2 items or unclear risk of bias ≥3 items). One of them [52] did not assess dietary intake and physical activity, whilst two of them [50,51] did not assess physical activity during the intervention. This may result in non-reliable conclusions because possible changes in physical activity or/and dietary intake throughout the period of intervention may influence the above metabolic parameters. In our view, conclusions of this systematic review should include and interpret only results from high-quality studies that assessed dietary intake and physical activity and did not demonstrate any significant differences in these parameters between baseline and post-intervention. Only five studies [46,47,48,49,56] in our search met these standards. 

Ebrahimi and his colleagues [46] only found a significant difference in DBP and not in SBP after supplementation of 100 mg saffron versus placebo in DM-2 patients for 12weeks. In another paper, the same investigators [47] did not find any significant difference in lipid profile and glycemic control but found significant difference in weight and WC. This may be explained by the findings from Gout et al. [58], who administrated 176.5 mg of saffron extract to mildly overweight healthy women and found a reduction of snacking and longer lasting satiety which might have contributed to weight loss. Karimi et al., found a significant difference in FBG and HbA1c in prediabetic overweight/obese patients after supplementation with 15mg saffron for eight weeks, but no overall effect on the lipid profile. Furthermore, Milajerdi et al. [48,49], found no significant differences in blood pressure, lipid profile and HbA1c, but FBG was significantly decreased in the intervention arm after administration of 30 mg saffron extract in DM-2 patients for eight weeks. This agrees with the findings of a meta-analysis from Pourmasoumi et al. [59] that reports no clinical benefit on the lipid profile, but a beneficial effect of saffron on FBG and HbA1c. Our qualitative findings are in accordance with their conclusions, although in their analysis they did not focus on DM and MS and also included healthy subjects and populations with other diseases. Furthermore, they did not include six studies [46,47,50,52,55,56] that we considered in this review.

The findings presented in this systematic review are, in part, not in line with previous research in this area with animal models. There is abundant evidence in vitro and in vivo animal studies that supplementation of saffron and its bioactive ingredients have a beneficial effect on the lipid profile and blood pressure [17,18,19,20,21,22,23,24,25,26]. Potential mechanisms of the effect on the lipid profile may be a potential inhibitory action of saffron and its bioactive ingredients on pancreatic lipase [60], antioxidant action, increase of the levels of adiponectin, activation of peroxisome proliferator-activated receptor alpha (PPARα)and modulation of heat shock proteins. Hypotensive effects can be attributed to blocking of calcium channels and possible interaction with endothelial nitric oxide (NO) [61]. Our findings do not match the results from animal studies, possibly due to varying doses and small duration of administration in humans. Nevertheless, regarding FBG, evidence from animal models [8,9,10,11,12,13] is in accordance with our findings on humans [47,56].

It is plausible that a number of limitations might have influenced the results of this systematic review. As mentioned above, there is a limited number of high-quality studies examining the effects of saffron on humans without major methodological issues. There is high heterogeneity in the studiesincluded, mainly regarding the amount and form of supplement used. As Tomé-Carneiro et al. [62] mentioned, a significant parameter in nutraceutical research is bioavailability. Different preparation methods do not supply an obtainable form of nutraceuticals. There is no clear evidence on the bioavailability and absorption in the gastrointestinal tract of saffron and its bioactive constituents. From what is shown in recent bibliography, antioxidant supplements do not work exactly how we were hoping. As has been shown in a systematic review and meta-analysis of 68 RCTs [63] examining the effect of antioxidant supplements on mortality, primary and secondary prevention of diseases, supplements are not effective in reducing oxidative stress and mortality. Although in vitro trials show the effectiveness of antioxidants, this does not apply in vivo as it has been shown that antioxidant molecules are not effective, due to kinetic constraints and limited bioavailability [64]. Another limitation of the current literature review is that there is no report of titration in the administered supplements. Additionally, there is limited evidence of the impact of saffron and its constituents on the metabolic profile in patients with DM and MS because the existing studies come from a limited number of research groups with few different study protocols, despite the number of published papers. 

Moreover, the study populations are only Caucasians, an important limitation, because the etiology of DM2 differs among ethnic groups. Furthermore, another limitation is that we included study populations with DM and MS which were analyzed as one group in this review, not considering β- cell dysfunction as a pathophysiological mechanism of DM, like insulin resistance [65].

Our study emphasizes on patients with impaired glucose tolerance and DM, but in the existing literature we found scarce evidence and there is high heterogeneity in the results of clinical trials. Therefore, we did not proceed to quantitative analysis and meta-analysis like Pourmasoumi but assessed more qualitatively existing trials.

To the best of our knowledge, this is the first systematic review investigating the impact of saffron on metabolic parameters in patients with DM and MS. One strength of our review is to focus only on patients with DM and MS. Another strength is that we included in the interpretation of our results the information whether dietary and physical activity (being two important cofounders) assessments were performed during the intervention period.

We propose that there is a need to conduct more high-quality clinical trials with different ethnic groups in order to investigate the potential beneficial impact of saffron supplementation on glycemic control and lipid profile of DM and MS patients in order to establish whether saffron could be a possible adjunct to diabetes therapy.

## 5. Conclusions

In summary, findings from this review are implausible due to the low-quality clinical trials assessed. It may be a favorable effect of saffron in FBG, but further research needs to be carried out in populations with greater homogeneity, different ethnic groups, more particular doses, and duration of supplementation. Also, it is necessary for the titration of the supplement used to provide more consistent results.

## Figures and Tables

**Table 1:** Summary of study characteristics.

Study (Year)	Sample	Gender (Males)	Age (Years)	Study Design	Condition	Intervention/Groups	Duration	Outcome	Assessment of Diet	Assessment of Physical Activity
**Abedimanesh 2017** [57]	75	14/13/12	56.04 ± 7.55 53.36 ± 5.94 56.32 ± 5.91	Randomized Double blind placebo controlled	Coronary artery disease (17% DM)	Saffron aqueous extract (30 mg) vs. Crocin (30 mg) vs. Placebo	8 weeks	FBG, t-chol, HDL-c, LDL-c, TG, WC	Yes	No
**Azimi 2014** [44]	208	17/16/15/16/15	54.33 ± 0.5	Parallel Randomized Single Blind Placebo controlled	DM-2	3 gl of Black tea + 3 g cardamom vs. 3 gl black tea + 3 g cinnamon 3 gl Black tea + 3 g ginger vs. 3 gl black tea + 1 g saffron vs. 3 gl black tea (control)	8 weeks	FBG, t-chol, TG, LDL-c, HDL-c, HbA1c,	Yes	Yes
**Azimi 2016** [45]	208	17/16/15/16/15	54.33 ± 0.5	Parallel Randomized Single Blind Placebo controlled	DM-2	3 gl of black tea + 3 g cardamom vs. 3 gl black tea + 3 g cinnamon vs. 3 gl black tea + 3 g ginger vs. 3 gl black tea + 1 g saffron vs. 3 gl black tea (control)	8 weeks	WC, SBP, DBP	Yes	Yes
**Ebrahimi 2019** [46]	90	36	55.2 ± 7.3 53 ± 10.6	Prospective DoubleBlind Placebo Controlled Randomized	DM-2	Saffron 100 mg vs. placebo 100 mg maltodextrin	12 weeks	SBP, DBP	Yes	Yes
**Ebrahimi 2019b** [47]	90	36	55.2 ± 7.3 53 ± 10.6	Prospective Double Blind Placebo Controlled Randomized	DM-2	Saffron 100 mg vs. placebo 100 mg maltodextrin	12 weeks	FBG, HbA1c, TG, t-chol, HDL-c, LDL-c, WC	Yes	Yes
**Javandoost 2017** [52]	44	18	44.50 (24.75–51.50) 33.10 (29.85–35.42)	Double blind randomized placebo controlled	MS	30 mg crocin vs. placebo	8 weeks	FBG, TG, HDL-c, LDL-c, t-chol	No	No
**Karimi Nazari2019** [56]	80	27	57.95 ± 8.18 57.9 ± 8.7	Double blind randomized placebo controlled	Prediabetes	15 mg saffron vs. placebo	8 weeks	FBG, TG, HDL-c, LDL-c, t-chol	Yes	Yes
**Kermani 2017** [53]	48	7	53.8 ± 9.2 50.9 ± 8.8	Double blind Randomized placebo controlled	MS	100 mg crocin vs. placebo	6 weeks	FBG, TG, HDL-c, LDL-c, t-chol, SBP, DBP, WC	No	No
**Milajerdi 2017** [48]	54	12	54.57 ± 6.96 55.42 ± 7.58	Double blind Randomized placebo controlled	DM-2	30 mg saffron vs. placebo	8 weeks	WC, SBP, DBP (does not report full data)	Yes	Yes
**Milajerdi 2018** [49]	54	12	54.57 ± 6.96 55.42 ± 7.58	Triple blind randomized placebo controlled	DM-2	30 mg saffron vs. placebo	8 weeks	FBG, t-chol, TG, HDL-c, LDL-c, HbA1c	Yes	Yes
**Moravej Aleali 2019** [50]	64	19	52.4 ± 13 53.5 ± 9.9	Double blind Randomized placebo controlled	DM-2	30 mg saffron vs. placebo	3 months	FBG, t-chol, TG, HDL-c, LDL-c, HbA1c,	Yes	No
**Nikbakht-Jam 2016** [54]	60	25	38.97 ± 13.33 43.46 ± 12.77	Double blind Randomized placebo controlled	MS (DM 16%)	30 mg crocin vs. placebo	8 weeks	FBG, t-chol, TG, HDL-c, LDL	No	No
**Zilaee 2018** [55]	76	9	42.19 ± 11.52 43.60 ± 9.05	Double blind Randomized placebo controlled	MS	100 mg saffron vs. placebo	12 weeks	LDL-c, HDL-c, TG, t-chol, WC	No	Yes (self-reported)
**Sepahi 2018** [51]	60	29	53.31 ± 6.6 56.09 ± 4.3 57.17 ± 2.9	Double masked Randomized phase 2 placebo controlled	DM-1:10 DM-2:50	Crocin 5 mg vs. crocin 15 mg vs. placebo	3 months	FBG, HbA1c HDL-c, LDL-c, TG, t-Chol	No	No

Abbreviations used: DM-2 (diabetes mellitus type 2), FBG (fasting blood glucose), t-chol (total cholesterol), HDL-c (high-density lipoproteincholesterol), LDL-c (low-density lipoproteincholesterol), MS (metabolic syndrome), TG (triglycerides), WC (waist circumference), HbA1c (glycated haemoglobin), SBP (systolic blood pressure), DBP (diastolic blood pressure), gl (glasses).

**Table 2:** Summary of findings for fasting blood glucose (FBG).

Study Name, Year		Intervention Group					Control Group				
		Baseline		Post-Intervention			Baseline		Post-Intervention		
	*N*	Mean	SD	Mean	SD	*N*	Mean	SD	Mean	SD	*p*
Abedimanesh 2017 # [57]	25	107.25	44.14	100.85	26.49	25	123.11	59.31	117.63	56.48	NS
Azimi 2014 # [44]	42	358.35	4.3	356.66	4.39	39	355.28	11.86	353.23	11.96	NS
Ebrahimi 2019b [47]	40	166.7	53.7	162.1	52.7	40	160.9	51.9	147.7	51.8	NS
Karimi Nazari 2019 [56]	36	118.11	3.55	109.14	6.23	39	119.15	4.03	118.87	6.27	0.005
Javandoost 2017 [52]	21	94.08	16.49	91	20.67	22	102.75	27.5	103.83	23.77	NS
Kermani 2017 # [53]	24	110	42.16	111.2	35.6	24	124.4	47.7	129.3	75	NS
Milajerdi 2018 [49]	26	164.36	40.88	128.84	31.86	26	159.64	38.38	153.76	41.23	<0.001
Moravej Aleali 2019 [50]	32	173.2	73.9	147.9	53.5	32	177.1	60.1	188.5	74.7	0.013
Nikbakht-Jam 2016 # [54]	30	102.34	36.88	104.52	49.2	30	101.31	29.08	103.31	25.18	NS
Sepahi 2018 [51]	55	176.6	64.17	155.39	55.49	23	175.15	7.38	169.45	7.61	NS

Abbreviations used: SD, standard deviation; NS (not significant), *p* < 0.05 was considered statistically significant. **#** study of low quality according to risk of bias assessment.

**Table 3:** Summary of findings for glycated hemoglobin(HbA1c).

Study Name, Year		Intervention Group					Control Group				
		Baseline		Post-Intervention			Baseline		Post-Intervention		
	*N*	Mean	SD	Mean	SD	*N*	Mean	SD	Mean	SD	*p*
Azimi 2014 # [44]	42	7.73	0.07	7.74	0.07	39	7.5	0.1	7.51	0.1	NS
Ebrahimi 2019b [47]	40	8.01	1.4	7.69	1.49	40	7.38	1.53	7.34	1.48	NS
Karimi Nazari 2019 [56]	36	5.85	0.12	5.7	0.11	39	5.88	0.11	5.92	0.12	<0.005
Milajerdi 2018 [49]	26	6.37	1.3	6.75	1.28	26	6.83	1.36	7.25	1.65	NS
Moravej Aleali 2019 [50]	32	8.9	2	8.2	1.8	32	8.8	1.8	8.3	1.4	NS
Sepahi 2018 [51]	23	8.17	0.11	7.29	0.12	23	8.15	0.22	8.03	0.14	0.024 *

Abbreviations used: SD, standard deviation; NS (not significant), *p* < 0.05 was considered statistically significant. # study of low quality according to risk of bias assessment. * This study had one more intervention group (5 mg crocin) that did not show a significant difference compared to the placebo group.

**Table 4:** Summary of findings for total cholesterol (t-chol).

Study Name, Year		Intervention Group					Control Group				
		Baseline		Post-Intervention			Baseline		Post-Intervention		
	*N*	Mean	SD	Mean	SD	*N*	Mean	SD	Mean	SD	*p*
Azimi 2014 # [44]	42	395	212	394.3	2.22	39	338.92	8.78	334.92	8.87	0.004
Ebrahimi 2019b [47]	40	143.7	36.6	152.8	31.4	40	147	32.5	155.1	37.2	NS
Karimi Nazari 2019 [56]	36	186.67	17.22	184.54	17.45	39	192.69	13.57	190.88	14.6	NS
Javandoost 2017 [52]	22	232.18	66.52	220.09	55.6	22	209.19	38.41	199.95	50.1	NS
Kermani 2017 # [53]	24	230.1 ^a^	42.3	204.5 ^a^	41.2	24	232.2 ^b^	49.7	208.6 ^b^	41	NS
Milajerdi 2018 [49]	26	179.04	35.29	166.96	25.8	26	181.44	33.19	169.28	25.57	NS
Moravej Aleali 2019 [50]	32	169.3	38.8	152.9	32.1	32	152.21	31.5	164.2	43.5	0.014
Nikbakht-Jam 2016 # [54]	30	224.48	60.83	210.52	52.68	30	212.76	37.82	210.9	50.3	NS
Sepahi 2018 [51]	55	196.54	55.2	199.02	49.5	23	189.45	7.24	190.85	7.17	NS
Zilaee 2018 # [55]	30	199.15	27.3	96.88	37.73	31	177.16	33.34	167.36	37.03	NS

Abbreviations used: NS (not significant), *p* < 0.05 was considered statistically significant, ^a^ (*p* < 0.001), ^b^ (*p* = 0.01). # study of low quality according to risk of bias assessment.

**Table 5:** Summary of findings for LDL cholesterol (LDL-c).

Study Name, Year		Intervention Group					Control Group				
		Baseline		Post-Intervention			Baseline		Post-Intervention		
	*N*	Mean	SD	Mean	SD	*N*	Mean	SD	Mean	SD	*p*
Abedimanesh 2017 # [57]	25	94.1	35.4	89.2	32.34	25	81.31	28.47	83.21	26.23	NS
Azimi 2014 # [44]	42	229.57	2.64	228.28	2.63	39	208.64	6.06	205.94	5.51	0.01
Ebrahimi 2019b [47]	40	82.7	25.7	89.5	23.9	40	84.5	26.6	90.7	30.5	NS
Karimi Nazari 2019 [56]	36	114.75	13.25	113.55	12.77	39	120.31	12.69	117.72	11.34	NS
Javandoost 2017 [52]	21	162.67	66.78	131.25	54.66	22	130.92	39.8	116.17	70.5	NS
Kermani 2017 # [53]	24	146.3	25.4	139.1	25.8	24	147.2	44.9	127.5	32.1	NS
Milajerdi 2018 [49]	26	83.79	29.48	85.9	32.04	26	95.9	36.16	82.94	26.95	NS
Moravej Aleali 2019 [50]	32	87.7	26.1	72.9	26.2	32	81.2	25.4	82.9	40.5	0.014
Nikbakht-Jam 2016 # [54]	30	152.29 ^a^	56.93	123.52 ^a^	48.06	30	138.45	36.76	125.76	52.16	NS
Sepahi 2018 [51]	55	120.99	43.15	118.54	46.13	23	113.85	6.02	110.45	5.31	NS
Zilaee 2018 # [55]	30	120.03	30.01	97.65	25.88	31	125.16	22.33	113	26.56	0.03

Abbreviations used: *NS* (not significant), *p* < 0.05 was considered statistically significant, ^a^ (*p* = 0.02). # study of low quality according to risk of bias assessment.

**Table 6:** Summary of findings for HDL cholesterol (HDL-c).

Study Name, Year		Intervention Group					Control Group				
		Baseline		Post-Intervention			Baseline		Post-Intervention		
	*N*	Mean	SD	Mean	SD	*N*	Mean	SD	Mean	SD	*p*
Abedimanesh 2017 # [57]	25	42.35	6.74	45.7	9.06	25	45.84	6.52	47.84	8.33	NS
Azimi2014 # [44]	42	53.97	0.71	54.76	0.74	39	50.38	1.38	51.53	1.52	0.001
Ebrahimi 2019b [47]	40	41.8	8.4	42.2	9.4	40	44.35	10.1	44.9	11.3	NS
Karimi Nazari 2019 [56]	36	49.97	11.62	50.25	11.15	39	52.2	8.8	52	9.8	NS
Javandoost 2017 [52]	21	38.25 ^a^	11.33	48.92 ^a^	12.5	22	38.17 ^b^	10.7	52.5 ^b^	15.06	NS
Kermani 2017 # [53]	24	40.3	8.4	40	7.8	24	38.4	6.8	38.6	7.3	NS
Milajerdi 2018 [49]	26	58.83	8.47	63.33	5.11	26	60.95	7.17	6.17	7.08	NS
Moravej Aleali 2019 [50]	32	45.1	9.1	48.2	10.6	32	38.3	9.6	43	11.1	NS
Nikbakht-Jam 2016 # [54]	30	38.59	10.14	49.25	11.5	30	38.93	9.18	51.24	10.44	NS
Sepahi 2018 [51]	55	43.57	11.53	42.94	10.3	23	43.95	0.94	44.35	0.85	NS
Zilaee 2018 # [55]	30	39.03	5.34	43	9.97	31	39.13	8	43.46	6.5	NS

Abbreviations used: NS (not significant), *p* < 0.05 was considered statistically significant, ^a^ (*p* = 0.004), ^b^ (*p* < 0.001). # study of low quality according to risk of bias assessment.

**Table 7:** Summary of findings for triglycerides (TG).

Study Name, Year		Intervention Group						Control Group				
		Baseline		Post-Intervention				Baseline		Post-Intervention		
	*N*	Mean	SD	Mean	SD	*N*	Mean		SD	Mean	SD	*p*
Abedimanesh 2017 # [57]	25	200.05	74.08	193.05	60.44	25	182.37		87.27	192.32	101	NS
Azimi 2014 # [44]	42	391.88	3.91	390.71	3.82	39	386.54		13.28	382.48	12.72	NS
Ebrahimi 2019b [47]	40	165.8	121.8	175	98.1	40	170.4		63.5	168	58.3	NS
Karimi Nazari 2019 [56]	36	101.5	20.34	100.22	17.63	39	108.94		18.2	107.84	15.97	NS
Javandoost 2017 [52]	21	155.58	73.74	163.25	99.58	22	164.33		84.8	153.67	75.29	NS
Kermani 2017 # [53]	24	218.1^a^	80	173.8^a^	97.5	24	232.4		83.2	197.5	82.9	NS
Milajerdi 2018 [49]	26	146.54	41.86	127	37.61	26	137.96		40.71	128.2	38.5	NS
Moravej Aleali 2019 [50]	32	166.4	87.7	156.4	73.2	32	187.2		137.1	191.8	135.3	NS
Nikbakht-Jam 2016 # [54]	30	153.17	67.06	147	72.52	30	165.47		76.73	153.9	89.9	NS
Sepahi 2018 [51]	55	199.65	112.24	196.32	97.6	23	203.75		9.32	200.7	8.09	NS
Zilaee2018 # [55]	30	139,76	70,14	96,88	37,73	31	139		73,52	107,6	43,98	NS

Abbreviations used: NS (not significant), *p* < 0.05 was considered statistically significant, ^a^ (*p* < 0.003). # study of low quality according to Risk of Bias Assessment.

**Table 8:** Summary of findings for systolic blood pressure (SBP).

Study Name, Year		Intervention Group					Control Group				
		Baseline		Post-Intervention			Baseline		Post-Intervention		
	*N*	Mean	SD	Mean	SD	*N*	Mean	SD	Mean	SD	*p*
Azimi 2016 # [45]	42	139.08	0.2	139	0.1	39	136.9	0.2	137.2	0.2	NS
Ebrahimi 2019 [46]	40	132.7	21.3	124.5	13.2	40	127.4	15.3	128.3	12.4	0.005
Kermani 2017 # [53]	24	129.3	16.9	126.8	19.4	24	131	14	131.8	13.5	NS

Abbreviations used: NS (not significant), *p* < 0.05 was considered statistically significant. # study of low quality according to risk of bias assessment.

**Table 9:** Summary of findings for diastolic blood pressure (DBP).

Study Name, Year		Intervention Group					Control Group				
		Baseline		Post-Intervention			Baseline		Post-Intervention		
	*N*	Mean	SD	Mean	SD	*N*	Mean	SD	Mean	SD	*p*
Azimi 2016# [45]	42	94.06	0.1	94.02	0.1	39	92.7	0.1	93.9	0.1	NS
Ebrahimi 2019 [46]	40	79.5	10.8	76.7	9.9	40	79.7	11.1	75.9	14	NS
Kermani 2017 # [53]	24	81.1	12.8	80.9	14.5	24	85	20.6	84.1	13.4	NS

Abbreviations used: NS (not significant), *p* < 0.05 was considered statistically significant. # study of low quality according to risk of bias assessment.

**Table 10:** Summary of findings for waist circumference (WC).

Study Name, Year		Intervention Group					Control Group				
		Baseline		Post-Intervention			Baseline		Post-Intervention		
	*N*	Mean	SD	Mean	SD	*N*	Mean	SD	Mean	SD	*p*
Abedimanesh 2017 # [57]	25	95	12.85	92.68	13.03	25	92.84	9.13	91.42	8.94	NS
Azimi 2016 # [45]	42	102.26	1.5	102.02	1.5	39	100.92	1.4	100.66	1.4	NS
Ebrahimi 2019b [47]	40	104.31	7.85	100.02	7.32	40	102.95	7.94	104.33	7.42	<0.001
Kermani2017 # [53]	24	103.9	9.5	103.2	9	24	101.5	8.1	105.9	15.7	NS
Zilaee 2018 # [55]	32	105.76	9.01	103	9.02	32	103.36	12.09	101.03	12.69	NS

Abbreviations used: NS (not significant), *p* < 0.05 was considered statistically significant. # study of low quality according to risk of bias assessment.

## Supplementary Materials

The following are available online at https://www.mdpi.com/2072-6643/12/5/1424/s1, PPRISMA 2009 Checklist.

## Abbreviations

AGEs	advanced glycation end product
PPARa	peroxisome proliferator-activated receptor alpha
BP	blood pressure
CK	creatine kinase
DM	Diabetes mellitus
FBG	fasting blood glucose
HbA1c	Glycatedhaemoglobin
HDL-C	high-density cholesterol
LDH	levels of lactate dehydrogenase
LDL-C	low-density cholesterol
MDA	malondialdehyde
MS	metabolic syndrome
NO	nitric oxide
TG	triglycerides
SOD	superoxide dismutase
t-chol	total serum cholesterol
WC	waist circumference
